# Special Issue “Advances in and Insights into the Treatment of Glaucoma”

**DOI:** 10.3390/ijms262412135

**Published:** 2025-12-17

**Authors:** Wei-Ting Yen, Da-Wen Lu

**Affiliations:** 1 Department of Ophthalmology, College of Medicine, National Defense Medical University, Taipei 11490, Taiwan; jimmyyen59@gmail.com; 2 Department of Ophthalmology, Tri-Service General Hospital, Taipei 11490, Taiwan

## 1. Introduction

Despite advances in detection and treatment, glaucoma still imposes a substantial global burden as a leading cause of permanent blindness, with approximately 111.8 million individuals projected to be affected by 2040 [1]. Although traditionally defined by intraocular pressure (IOP)-related damage to the optic nerve head, it is increasingly recognized as a multifactorial neurodegenerative disease involving complex crosstalk among biomechanical stress, vascular dysregulation, extracellular matrix (ECM) remodeling, immune activation, and retinal ganglion cell (RGC) vulnerability within the eye–brain axis [2,3]. Lowering IOP remains the only proven disease-modifying strategy in clinical practice, yet many patients continue to progress despite apparently adequate IOP control, underscoring the need for therapies that directly target neurodegeneration, fibrotic wound healing, and aging-related biology alongside aqueous humor dynamics [4,5,6]. Against this backdrop, this Special Issue, “Advances In and Insights into the Treatment of Glaucoma”, brings together six mechanistically oriented contributions that span Rho kinase (ROCK) inhibition, MicroPulse transscleral laser therapy, filtration surgery fibrosis, ECM genetics, accelerated biological aging in exfoliation glaucoma (EXG), and endoplasmic reticulum (ER) stress-driven RGC loss. Collectively, these articles illustrate how molecular and imaging insights can refine current treatment paradigms and point toward future disease-modifying strategies.

## 2. Highlights of the Contributions

### 2.1. ROCK Inhibitors: Multifaceted Mechanisms and Therapeutic Potential

Liu and colleagues provide a comprehensive review, which synthesizes preclinical and clinical evidence on topical ROCK inhibitors and their place in contemporary glaucoma care [7]. They emphasize how modulation of trabecular meshwork (TM) cytoskeletal tone and ECM architecture enables a mechanistically distinct IOP-lowering approach that complements prostaglandin analogues and aqueous suppressants. Their review also highlights the pleiotropic actions of ROCK inhibitors—on ocular blood flow, inflammatory signaling, and post-trabeculectomy wound healing—that expand their relevance beyond simple hypotensive agents. These concepts extend and contextualize recent work on ROCK inhibitors as emerging targets that not only enhance conventional outflow but may also influence optic nerve perfusion and neuroinflammatory cascades [8,9,10], supporting the broader view that pathway-directed TM therapies could be combined with neuroprotective strategies to achieve more durable protection of visual function.

### 2.2. MicroPulse Transscleral Laser Therapy: Structural and Functional Evidence

Agnifili et al. focus on MicroPulse transscleral laser therapy (MicroPulse TLT) for refractory glaucoma, using anterior segment optical coherence tomography (AS-OCT) to visualize uveoscleral outflow routes following treatment [11]. By correlating scleral and uveoscleral structural changes with IOP outcomes, they provide rare in vivo anatomical evidence supporting the hypothesis that MicroPulse TLT can modulate outflow beyond purely cyclodestructive mechanisms. Their findings complement long-term and real-world series demonstrating that MicroPulse transscleral cyclophotocoagulation can achieve clinically meaningful and sustained IOP reductions with a more favorable safety profile than continuous-wave cyclodestructive procedures, particularly with respect to inflammation, hypotony, and visual acuity loss [12,13]. Together, these data support positioning MicroPulse TLT as a flexible option across the glaucoma severity spectrum, especially in eyes where conjunctival preservation or prior filtration surgery complicates further incisional interventions.

### 2.3. Filtration Surgery Fibrosis: Molecular Drivers and Antifibrotic Strategies

Sacchi and co-authors provide an in-depth overview of the cellular and molecular events that underlie filtration bleb failure [14]. They delineate how TGF-β/Smad signaling, connective tissue growth factor, matricellular proteins, and inflammatory cytokines orchestrate conjunctival and tenon’s capsule fibroblast activation, ECM deposition, and angiogenesis at the bleb site, and discuss how these pathways intersect with systemic fibrotic disorders. Their review critically appraises current antimetabolite-based modulation and then pivots toward emerging targeted approaches, including ROCK inhibition, anti-TGF-β strategies, and agents aimed at ECM cross-linking and myofibroblast differentiation, which may offer more selective and safer control of postoperative wound healing [15,16]. By integrating mechanistic data from ocular and extra-ocular fibrosis models, this article underscores that future trabeculectomy and tube-shunt surgery could be coupled to pathway-specific antifibrotic regimens tailored to individual risk profiles rather than relying solely on empirical dosing of mitomycin C or 5-fluorouracil.

### 2.4. Genetic Variants in ECM Remodeling and Glaucoma Susceptibility

Atanasovska Velkovska et al. address the role of genetic variability in ECM remodeling in glaucoma by examining matrix metalloproteinase (MMP) polymorphisms and their associations with disease risk, clinical phenotype, and response to selective laser trabeculoplasty (SLT) versus latanoprost [17]. Their data show that while specific MMP genotypes influence glaucoma susceptibility and phenotype, they did not predict response to SLT or latanoprost, though the findings still offer an initial framework for exploring ECM-based pharmacogenomic stratification in future therapy selection. These findings regarding MMP-mediated remodeling align with a broader genetic framework showing that ECM-related genes, including those regulating collagen assembly, elastogenesis, and ECM turnover, contribute to both primary open-angle glaucoma (POAG) and exfoliation-related disease [18,19,20,21]. LOXL1 polymorphisms, for example, have long been recognized as strong risk factors for exfoliation syndrome (XFS) and EXG [20], and large-scale GWAS analyses have refined their context among other loci that regulate ECM mechanics at the trabecular meshwork and lamina cribrosa [21]. Taken together, these findings underscore the central role of genetically regulated ECM biology in glaucoma susceptibility and in shaping how ocular tissues respond to IOP-lowering interventions.

### 2.5. Accelerated Biological Aging in Exfoliation Glaucoma

Tanito and Koyama explore the intriguing intersection between glaucoma and systemic biological aging in their study [22]. Using a deep learning-derived “predicted age” from color fundus photographs and systemic measures of advanced glycation end products (AGEs), they demonstrate that EXG eyes exhibit greater biological age acceleration than both POAG and non-glaucomatous controls, even after accounting for chronological age and conventional risk factors. Their findings align with emerging population-based data showing that biological age acceleration, estimated from multi-omic or clinical markers, is associated with subsequent glaucoma risk and may interact with genetic risk scores [23]. Earlier biochemical work on aqueous humor and systemic biomarkers in glaucoma, including oxidative stress markers, AGEs, and ECM turnover products, also supports the concept that glaucoma, particularly EXG, reflects a state of accelerated tissue aging rather than purely IOP-mediated optic neuropathy [24]. Conceptually, this positions EXG as a model disease in which vascular, ECM, and aging-related pathways converge, and it raises the possibility that interventions modulating systemic aging biology could one day complement ocular hypotensive and surgical treatments.

### 2.6. ER Stress-Induced Neurodegeneration and the Role of CHOP

Mayhew et al. complete the bench-to-bedside arc of this Special Issue by focusing on intrinsic neuronal stress pathways in RGCs. They show that RGC-specific expression of C/EBP homologous protein (CHOP), a key ER stress effector, is sufficient to drive structural and functional neurodegeneration in vivo [25]. Their work builds on earlier experimental glaucoma models in which chronic ocular hypertension triggered ER stress and CHOP upregulation in RGCs and the optic nerve, leading to apoptotic cell death [26], and it dovetails with recent interventional studies, demonstrating that genetic or pharmacologic disruption of the ATF4–CHOP axis can rescue RGC survival and preserve visual function in ocular hypertension and optic nerve crush paradigms [27]. Together, these studies strengthen the case for ER stress signaling as a tractable neuroprotective target in glaucoma and illustrate how mechanistic insights from basic science can be translated into concrete therapeutic hypotheses, such as small-molecule ER stress modulators, gene therapy, or cell-intrinsic resilience boosters, which may eventually be combined with IOP-lowering and antifibrotic strategies.

## 3. Perspectives and Future Directions

Taken together, the six contributions in this Special Issue demonstrate how glaucoma treatment is shifting from a pressure-centric paradigm toward a multidimensional, mechanism-based strategy. ROCK inhibitors and MicroPulse TLT exemplify targeted modulation of aqueous outflow and ciliary body function; antifibrotic work on filtration surgery underscores the importance of rationally designed wound-healing modulation; ECM genetics and aging studies highlight how inherited and acquired tissue properties shape both disease susceptibility and treatment response; and ER stress research in RGCs points squarely at intrinsic neurodegenerative pathways that must be addressed if we are to achieve durable visual preservation. Future research will need to integrate these domains by (i) developing multimodal biomarkers that capture mechanical, vascular, inflammatory, and aging-related risk; (ii) designing clinical trials that embed mechanism-linked endpoints alongside standard visual function and IOP outcomes; and (iii) leveraging artificial intelligence and system-level modeling to predict individualized trajectories and therapeutic combinations. For clinicians, the message is that glaucoma management is poised to become more targeted, combinatorial, and biologically informed. For researchers, this Special Issue illustrates how mechanistic insights, ranging from ROCK signaling and ECM remodeling to ER stress-driven neurodegeneration and biological aging, can be translated into testable therapeutic strategies that bring us closer to genuine disease modification in glaucoma.

## References

[1] Tham Y.C., Li X., Wong T.Y., Quigley H.A., Aung T., Cheng C.-Y. (2014). Global prevalence of glaucoma and projections of glaucoma burden through 2040: A systematic review and meta-analysis. Ophthalmology.

[2] Weinreb R.N., Aung T., Medeiros F.A. (2014). The Pathophysiology and Treatment of Glaucoma: A Review. JAMA.

[3] Bou Ghanem G.O., Wareham L.K., Calkins D.J. (2024). Addressing neurodegeneration in glaucoma: Mechanisms, challenges, and treatments. Prog. Retin. Eye Res..

[4] Calkins D.J. (2012). Critical Pathogenic Events Underlying Progression of Neurodegeneration in Glaucoma. Prog. Retin. Eye Res..

[5] Khaw P.T., Bouremel Y., Brocchini S., Henein C. (2020). The Control of Conjunctival Fibrosis as a Paradigm for the Prevention of Ocular Fibrosis-Related Blindness: “Fibrosis Has Many Friends”. Eye.

[6] Williams P.A., Harder J.M., Foxworth N.E., Cochran K.E., Philip V.M., Porciatti V., Smithies O., John S.W.M. (2017). Vitamin B3 Modulates Mitochondrial Vulnerability and Prevents Glaucoma in Aged Mice. Science.

[7] Liu L.-C., Chen Y.-H., Lu D.-W. (2024). The application of Rho kinase inhibitors in the management of glaucoma. Int. J. Mol. Sci..

[8] Sugiyama T., Shibata M., Kajiura S., Okuno T., Tonari M., Oku H., Ikeda T. (2011). Effects of Fasudil, a Rho-Associated Protein Kinase Inhibitor, on Optic Nerve Head Blood Flow in Rabbits. Investig. Ophthalmol. Vis. Sci..

[9] Honjo M., Tanihara H., Kameda T., Kawaji T., Yoshimura N., Araie M. (2007). Potential Role of Rho-Associated Protein Kinase Inhibitor Y-27632 in Glaucoma Filtration Surgery. Investig. Ophthalmol. Vis. Sci..

[10] Serle J.B., Katz L.J., McLaurin E., Heah T., Ramirez-Davis N., Usner D.W., Novack G.D., Kopczynski C.C., ROCKET-1 and ROCKET-2 Study Groups (2018). Two Phase 3 Clinical Trials Comparing the Safety and Efficacy of Netarsudil to Timolol in Patients with Elevated Intraocular Pressure: Rho Kinase Elevated IOP Treatment Trial 1 and 2 (ROCKET-1 and ROCKET-2). Am. J. Ophthalmol..

[11] Agnifili L., Palamini A., Brescia L., Porreca A., Oddone F., Tanga L., Ruggeri M.L., Quarta A., Mastropasqua R., Di Nicola M. (2024). Uveoscleral outflow routes after MicroPulse laser therapy for refractory glaucoma: An optical coherence tomography study of the sclera. Int. J. Mol. Sci..

[12] Aquino M.C.D., Barton K., Tan A.M.W.T., Sng C., Li X., Loon S.C., Chew P.T.K. (2015). Micropulse versus Continuous Wave Transscleral Diode Cyclophotocoagulation in Refractory Glaucoma: A Randomized Exploratory Study. Clin. Exp. Ophthalmol..

[13] Sanchez F.G., Peirano-Bonomi J.C., Barbosa N.B., Khoueir Z., Grippo T.M. (2020). Update on Micropulse Transscleral Cyclophotocoagulation. J. Glaucoma.

[14] Sacchi M., Tomaselli D., Ruggeri M.L., Aiello F.B., Sabella P., Dore S., Pinna A., Mastropasqua R., Nubile M., Agnifili L. (2025). Fighting bleb fibrosis after glaucoma surgery: Updated focus on key players and novel targets for therapy. Int. J. Mol. Sci..

[15] Seibold L.K., Sherwood M.B., Kahook M.Y. (2012). Wound Modulation after Filtration Surgery. Surv. Ophthalmol..

[16] Shao C.G., Sinha N.R., Mohan R.R., Webel A.D. (2023). Novel Therapies for the Prevention of Fibrosis in Glaucoma Filtration Surgery. Biomedicines.

[17] Atanasovska Velkovska M., Goričar K., Blagus T., Dolžan V., Cvenkel B. (2024). Association of matrix metalloproteinases polymorphisms with glaucoma risk, glaucoma phenotype, and response to treatment with selective laser trabeculoplasty or latanoprost. Int. J. Mol. Sci..

[18] Wiggs J.L., Pasquale L.R. (2017). Genetics of Glaucoma. Hum. Mol. Genet..

[19] Gharahkhani P., Jorgenson E., Hysi P., Khawaja A.P., Pendergrass S., Han X., Ong J.S., Hewitt A.W., Segrè A.V., Rouhana J.M. (2021). Genome-Wide Meta-Analysis Identifies 127 Open-Angle Glaucoma Loci with Consistent Effect across Ancestries. Nat. Commun..

[20] Li X., He J., Sun J. (2021). LOXL1 gene polymorphisms are associated with exfoliation syndrome/exfoliation glaucoma risk: An updated meta-analysis. PLoS ONE.

[21] Aung T., Ozaki M., Lee M.C., Schlötzer-Schrehardt U., Thorleifsson G., Mizoguchi T., Igo R.P., Haripriya A., Williams S.E., Astakhov Y.S. (2017). Genetic Association Study of Exfoliation Syndrome Identifies a Protective Rare Variant at *LOXL1* and Five New Susceptibility Loci. Nat. Genet..

[22] Tanito M., Koyama M. (2025). Accelerated Biological Aging in Exfoliation Glaucoma Assessed by Fundus-Derived Predicted Age and Advanced Glycation End Products. Int. J. Mol. Sci..

[23] Song W.-Q., Zhong W.-F., Li Z.-H., Liu D., Ren J.-J., Shen D., Gao J., Chen P.-L., Yang J., Wang X.-M. (2025). Biological Age Acceleration, Genetic Susceptibility, and Incident Glaucoma Risk. Investig. Ophthalmol. Vis. Sci..

[24] Schlötzer-Schrehardt U., Naumann G.O.H. (2006). Ocular and Systemic Pseudoexfoliation Syndrome. Am. J. Ophthalmol..

[25] Mayhew W.C., Kaipa B.R., Li L., Maddineni P., Sundaresan Y., Clark A.F., Zode G.S. (2025). C/EBP Homologous Protein Expression in Retinal Ganglion Cells Induces Neurodegeneration in Mice. Int. J. Mol. Sci..

[26] Hu Y., Park K.K., Yang L., Wei X., Yang Q., Cho K.-S., Thielen P., Lee A.-H., Cartoni R., Glimcher L.H. (2012). Differential Effects of Unfolded Protein Response Pathways on Axon Injury-Induced Death of Retinal Ganglion Cells. Neuron.

[27] Zode G.S., Sharma A.B., Lin X., Searby C.C., Bugge K., Kim G.H., Clark A.F., Sheffield V.C. (2014). Ocular-Specific ER Stress Reduction Rescues Glaucoma in Murine Glucocorticoid-Induced Glaucoma. J. Clin. Investig..

